# A Comparative Analysis of Drinking Water Provision and Hepatitis A Incidence in Uzbekistan in 2010-2023

**DOI:** 10.7759/cureus.68347

**Published:** 2024-08-31

**Authors:** Jasur Juraev, Ulugbek Mirzaev, Ilkhom Juraev, Mirzarakhim Baynazarov, Botirjon Kurbanov

**Affiliations:** 1 Department of Preventive Services, Kyoto University, Kyoto, JPN; 2 Department of Epidemiology, Infectious Disease Control and Prevention, Graduate School of Biomedical and Health Sciences, Hiroshima University, Hiroshima, JPN; 3 Department of Traumatology and Orthopedics, Samarkand State Medical University, Samarkand, UZB; 4 Department of Epidemiology and Public Health, Sanitary-Epidemiological Welfare and Public Health Committee Under the Ministry of Health of the Republic of Uzbekistan, Tashkent, UZB

**Keywords:** uzbekistan, public health, hygiene, hepatitis a, drinking water

## Abstract

Objective

This study aims to analyze the relationship between access to safe drinking water and the incidence of hepatitis A in Uzbekistan from 2010 to 2023 to inform public health strategies for disease prevention.

Methods

We utilized hepatitis A incidence data from the Sanitary and Epidemiological Well-Being and Public Health Authority and drinking water provision data from the Government Statistics Agency of Uzbekistan. A linear regression analysis was performed using R 4.3.2 to investigate the correlation between these variables. The study examined hepatitis A cases per 100,000 population and the percentage of households with access to safe drinking water.

Results

Hepatitis A incidence fluctuated significantly over the study period, with a notable spike to 162 cases per 100,000 population in 2023, despite relatively stable access to safe drinking water (ranging from 67.4% to 77% of households). The analysis revealed a complex relationship between water access and hepatitis A incidence. The linear regression coefficient was 3.89 (adjusted R-squared: 0.3021, P-value: 0.02), indicating that each growing percent of water supply is raising the incidence of hepatitis 3.89 cases of hepatitis infection.

Conclusion

The reverse effect of water supply percentage and the incidence of hepatitis A incidence in Uzbekistan suggests that other factors play significant roles in disease transmission. These may include sanitation practices, hygiene behaviors, and vaccination coverage. The findings emphasize the need for a multifaceted approach to hepatitis A prevention, incorporating improved water infrastructure, enhanced sanitation, public education, and comprehensive vaccination programs. Further research is needed to identify specific determinants of hepatitis A transmission in Uzbekistan to guide targeted interventions and public health policies.

## Introduction

Hepatitis A is a viral liver disease that can cause mild to severe illness. The hepatitis A virus (RNA structure) was discovered in 1973 [1-3]. It is primarily transmitted through the fecal-oral route, often via the consumption of contaminated food or water.

In areas with poor sanitation and inadequate access to safe drinking water, hepatitis A can be endemic and pose a significant public health challenge that needs specific prevention measurements in the world. According to the World Health Organization, worldwide distribution causes about 1.5 million cases yearly [4,5].

The hepatitis A virus (HAV) is highly resistant to environmental conditions and can survive for extended periods in water and soil. It can withstand freezing and heat temperatures, remain infected for a few days to weeks in dried feces, and remain infectious for months in fresh and salt water. This resilience makes water a particularly effective vehicle for HAV transmission, especially in regions where water treatment and sanitation infrastructure are lacking [6-8].

Previous studies demonstrated that increasing water quality and quantity, sanitation, and hygiene lead to a decrease in HAV prevalence [9]. The risk of contracting hepatitis A is related to the availability of the following factors. HAV can persist in water sources for long periods, increasing the risk of outbreaks. In areas with poor sanitation, human waste can contaminate water supplies, creating a cycle of infection. Safe drinking water significantly reduces the risk of HAV transmission through water consumption. Clean water enables better personal and food hygiene practices, further reducing transmission risks.

The level of access to safe drinking water often correlates with overall public health infrastructure and disease prevention capabilities. Reducing hepatitis A incidence through improved water provision can lead to substantial savings in healthcare costs and improved productivity [10,11].

In Uzbekistan, a country that has faced challenges in water infrastructure and hepatitis A control, understanding the relationship between drinking water provision and disease incidence is crucial for public health planning and policy-making. While improving access to clean drinking water is critical, it should be complemented by vaccination strategies to achieve optimal public health outcomes. Implementation of routine and targeted vaccination programs along with ongoing efforts to improve water and sanitation quality can significantly reduce the incidence of hepatitis A in Uzbekistan. This study aimed to assess the relationship between access to safe drinking water and the incidence of hepatitis A in Uzbekistan and to show the importance of vaccination for hepatitis A.

## Materials and methods

**Figure 1:**
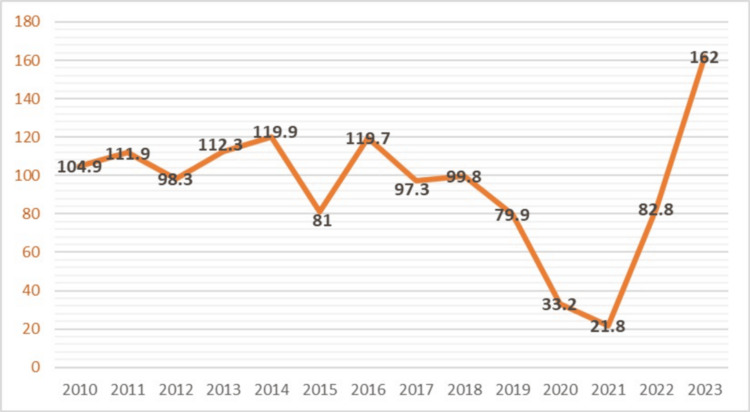
Hepatitis incidence from 2010 to 2023 in Uzbekistan

This retrospective study enrolled hepatitis A incidence data from 2010 to 2023 from the Sanitary and Epidemiological Well-Being and Public Health Authority under the Ministry of Health Republic of Uzbekistan. The variables included the incidence of hepatitis A cases per 100,000 population, categorized by regional distribution (Figure 1).

**Figure 2:**
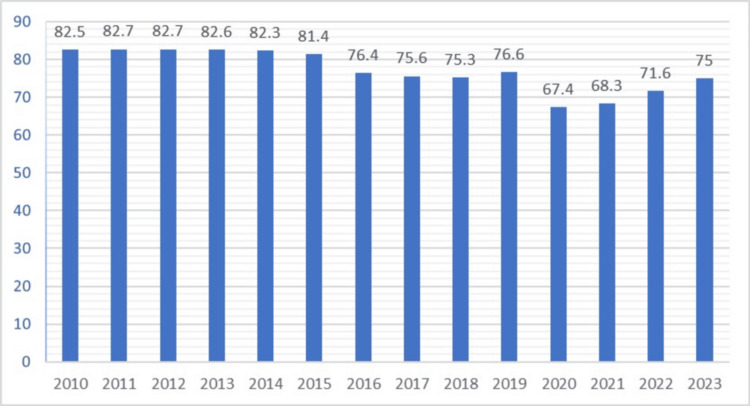
Providing drinking water to apartments from 2010 to 2023 in Uzbekistan

Drinking water provision data from 2010 to 2023 was downloaded from open data from the home page of the Statistics Agency under the President of the Republic of Uzbekistan. The variables included the percentage of households with access to safe drinking water and regional distribution of water infrastructure (Figure 2) [12].

Statistical analysis

For the statistical calculations, we used R 4.3.2 to perform a linear regression analysis of data on drinking water provision percentages and hepatitis A incidence in Uzbekistan from 2010 to 2023. Presentation of drinking water provision percentages and cases of hepatitis A was achieved using a frequency distribution in the form of n% and parametric data in the form of mean ± standard deviation (SD). The level of significance was set at P ≤ 0.05.

## Results

The analysis of the relationship between the percentage of households with access to safe drinking water and the incidence of hepatitis A in Uzbekistan from 2010 to 2023 reveals several key insights and raises important questions regarding public health and infrastructure.

Hepatitis A incidence trends

From 2010 to 2014 (Figure 1), the incidence of hepatitis A exhibited a fluctuating trend, ranging between 100 (82.5%) and 120 (82.3%) cases per 100,000 population. This period of relative instability could be attributed to various factors, including changes in public health policies, environmental conditions, and public awareness. The sharp decrease to 81 (81.4%) cases in 2015 might suggest the implementation of effective public health interventions or improvements in sanitation and hygiene practices during that year.

However, the subsequent increase to 120 (76.4%) cases in the following years indicates that the initial improvements were not sustained. This trend of fluctuating cases continued until 2021 when the incidence dropped significantly to 21.8 (68.3%) cases per 100,000 population. The sharp decline during this period may be partly attributed to the global COVID-19 pandemic, which led to enhanced hygiene practices, lockdowns, and reduced transmission of many infectious diseases, including hepatitis A.

The dramatic increase in hepatitis A cases to 162 (75%) per 100,000 population in 2023 is alarming. This surge could be due to several factors, including potential lapses in public health measures post-pandemic, changes in population behavior, or environmental factors. Further investigation is needed to identify the specific causes of this increase.

Access to safe drinking water

Throughout the same period (Figure 2), the percentage of households with access to safe drinking water remained relatively stable, with minor fluctuations. The rate decreased from 77% (n = 79.9) in 2019 to 67.4%-68.3% (n = 33.2-21.8) in 2020-2021, potentially due to disruptions caused by the COVID-19 pandemic. The gradual increase to 71% (n = 82.8) in 2022 and 75% (n = 162) in 2023 indicates ongoing efforts to improve water infrastructure.

Despite these efforts, the relatively stable access to safe drinking water does not seem to correlate directly with the fluctuations in hepatitis A incidence. This suggests that while access to safe drinking water is crucial, it may not be the sole determinant of hepatitis A incidence.

**Figure 3:**
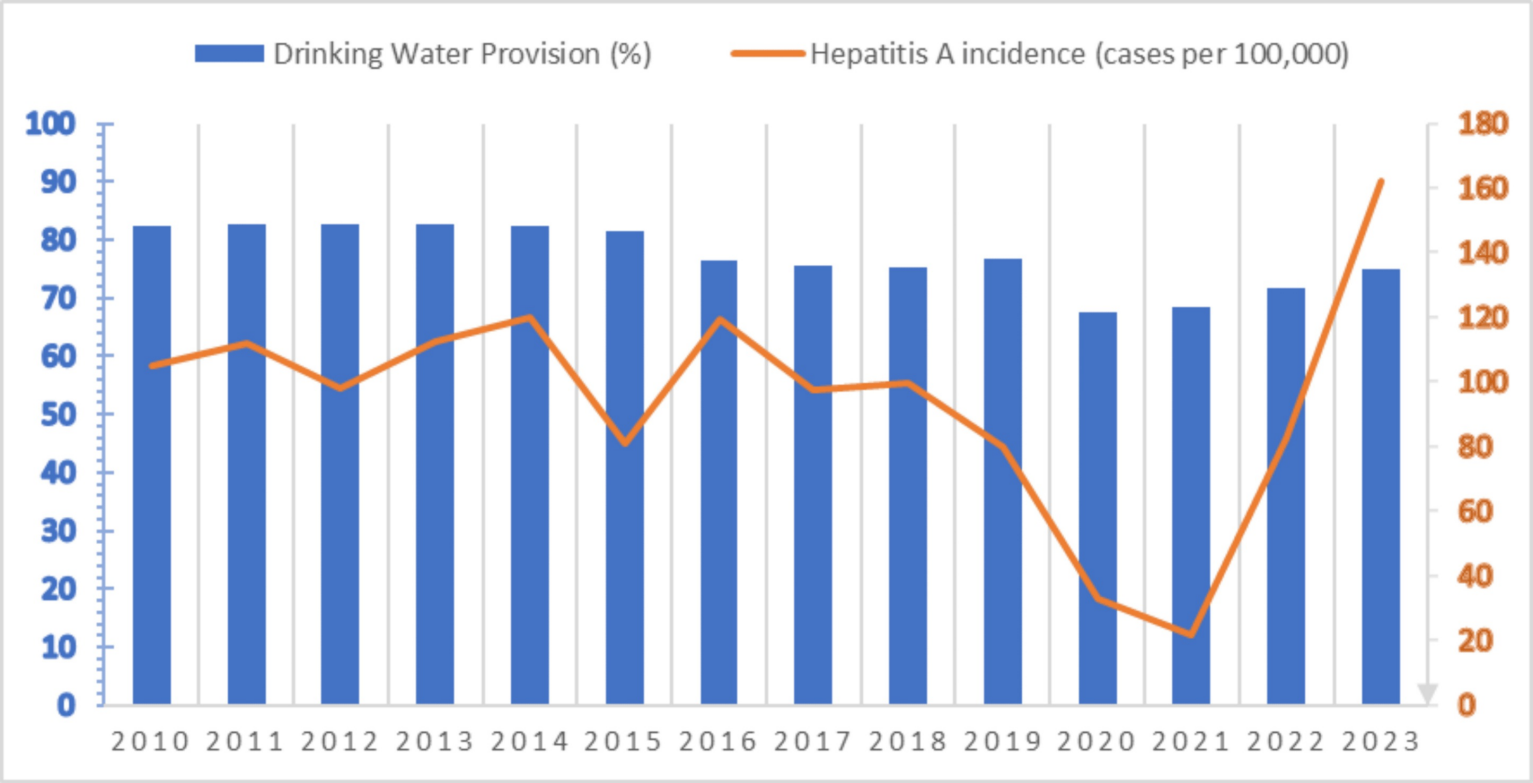
Comparison of the provision of drinking water in apartments and the incidence of hepatitis A (per 100,000 population) in 2010-2023 in Uzbekistan

This data (Figure 3) suggests that while drinking water provision may play a role in hepatitis A incidence, other factors likely influence the disease's prevalence, particularly in recent years. A sharp increase in 2022-2023 hepatitis A incidence is observed, despite the stable provision of drinking water. This suggests that the rise in hepatitis A cases cannot be solely explained by household water provision. The significant rise in hepatitis A cases during 2022-2023 could be related to pandemic-related factors. During the pandemic, people were largely confined to their homes, potentially leading to changes in hygiene practices or increased reliance on external sources of food and water, as workplaces and public areas reopened.

**Figure 4:**
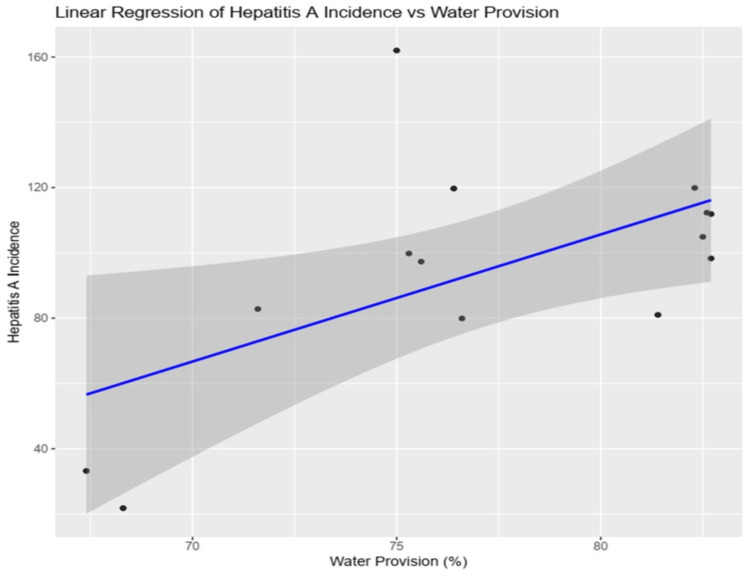
Linear regression of the provision of drinking water in apartments and the incidence of hepatitis A (per 100,000 population) in 2010-2023 in Uzbekistan

The scatter plot with a linear regression line (Figure 4) shows the relationship between water provision (in percentage) and hepatitis A incidence cases per 100 000 population, with a p-value of 0.02. The x-axis represents water provision, ranging from about 68% to 82%. The y-axis represents hepatitis A incidence, ranging from about 20 to 160. The slope suggests that an increase in drinking water provision is associated with an increase in hepatitis A incidence. However, the R² value shows that other factors also contribute significantly to the variability in hepatitis A incidence, and further investigation into those factors is necessary.

## Discussion

The findings of this study underscore the complexity of the relationship between access to safe drinking water and the incidence of hepatitis A in Uzbekistan. While improvements in water infrastructure are crucial, the data indicate that fluctuations in hepatitis A cases cannot be solely attributed to water access. The interesting part of this study is that in Uzbekistan, hepatitis A cases increased despite a rising percentage of households with access to safe drinking water. On the other hand, previous research demonstrated the opposite results, where hepatitis A decreased while the water supply increased in low- and middle-income countries [13,14]. These results suggest that other factors, such as sanitation, hygiene practices, and public health initiatives, likely play significant roles in influencing the incidence rates.

Sanitation and hygiene are critical components in preventing the transmission of hepatitis A, as the virus is primarily spread through the fecal-oral route [15,16]. Therefore, alongside efforts to improve water infrastructure, it is essential to enhance public awareness of proper hygiene practices, such as regular handwashing and safe food handling [17]. Public health campaigns focused on these areas could significantly reduce the spread of the virus, particularly in regions with limited access to safe drinking water [18,19].

Moreover, vaccination remains one of the most effective strategies to prevent hepatitis A. According to the World Health Organization (WHO), countries that have implemented widespread hepatitis A vaccination programs have seen substantial reductions in the incidence of the disease [14,20,21]. Incorporating hepatitis A vaccination into Uzbekistan’s national immunization schedule, particularly for children under the age of five, could provide long-term protection and reduce the burden of the disease. The resurgence of hepatitis A cases in 2023 further emphasizes the need for sustained vaccination efforts, even in the post-pandemic context [22,23].

Additionally, robust monitoring and surveillance systems are crucial for early detection and response to hepatitis A outbreaks. Such systems can help identify high-risk areas and populations, allowing for targeted interventions. Strengthening the country’s public health infrastructure, including laboratory capacity and disease reporting mechanisms, will be vital in mitigating future outbreaks [24-26].

Strengths of the study

No one has compared data in this manner before, and although the comparisons are very simple, they answer important questions about the relationship between hepatitis A and drinking water supply. The study covers 13 years (2010-2023), allowing for a comprehensive analysis of trends in both drinking water supplies and hepatitis A cases over time. This helps identify long-term patterns and fluctuations. The data are taken from official Uzbek sources providing information at the national level, increasing the generalizability of the results within the country.

Limitations

This study has several limitations that should be acknowledged. First, the analysis is based on aggregated data, which may obscure regional variations in water access and hepatitis A incidence. A more granular, region-specific analysis could provide additional insights into the relationship between these variables. Second, the study relies on secondary data, which may have inconsistencies or inaccuracies. The increase in hepatitis A cases after the pandemic may have been driven by people consuming food or water outside the home. The lack of detailed data on other potential confounding factors, such as individual hygiene practices or access to healthcare, limits our ability to fully understand the drivers of hepatitis A incidence. Finally, the study does not account for the possible impact of changes in public health policies, economic conditions, or environmental factors that could have influenced both water access and disease incidence over time.

## Conclusions

In conclusion, this study highlights the multifaceted nature of the relationship between safe drinking water provision and hepatitis A incidence in Uzbekistan. While access to clean water is undoubtedly a critical factor in preventing waterborne diseases, it is not the sole determinant of hepatitis A trends in the country. The fluctuations in hepatitis A cases observed over the study period suggest that other factors, such as sanitation, hygiene practices, vaccination coverage, and public health interventions, play significant roles in influencing disease incidence. To effectively reduce hepatitis A cases in Uzbekistan, a comprehensive approach is required. This should include sustained investments in water infrastructure, enhanced sanitation and hygiene promotion, and the integration of hepatitis A vaccination into the national immunization schedule.

Furthermore, strengthening public health surveillance and response systems will be essential in preventing future outbreaks and ensuring that interventions are targeted to the most vulnerable populations. There is much evidence and research that two doses of hepatitis A vaccine provide long-term, stable, significant protection and are cost-effective not only for patients but also for the government. The results of this study provide valuable information for public health policymakers and highlight the importance of a comprehensive approach to disease prevention. By addressing the multiple factors that contribute to hepatitis A transmission, Uzbekistan can make significant strides in reducing the burden of this preventable disease and improving overall public health outcomes.
